# Nur77 deletion impairs muscle growth during developmental myogenesis and muscle regeneration in mice

**DOI:** 10.1371/journal.pone.0171268

**Published:** 2017-02-07

**Authors:** Omar Cortez-Toledo, Caitlin Schnair, Peer Sangngern, Daniel Metzger, Lily C. Chao

**Affiliations:** 1 The Center for Endocrinology, Diabetes, and Metabolism, The Saban Research Institute, Children’s Hospital Los Angeles, Los Angeles, California, United States of America; 2 Institut de Génétique et de Biologie Moléculaire et Cellulaire, CNRS UMR7104/INSERM U964/Université de Strasbourg, Illkirch, France; 3 Department of Biochemistry & Molecular Biology, Keck School of Medicine, University of Southern California, Los Angeles, California, United States of America; University of Texas Health Science Center at Houston, UNITED STATES

## Abstract

Muscle atrophy is a prevalent condition in illness and aging. Identifying novel pathways that control muscle mass may lead to therapeutic advancement. We previously identified Nur77 as a transcriptional regulator of glycolysis in skeletal muscle. More recently, we showed that *Nur77* expression also controls myofiber size in mice. It was unknown, however, whether Nur77’s regulation of muscle size begins during developmental myogenesis or only in adulthood. To determine the importance of Nur77 throughout muscle growth, we examined myofiber size at E18.5, 3 weeks postnatal age, and in young adult mice. Using the global *Nur77*^*-/-*^ mice, we showed that Nur77 deficiency reduced myofiber size as early as E18.5. The reduction in myofiber size became more pronounced by 3 weeks of age. We observed comparable reduction in myofiber size in young myofiber-specific Nur77-knockout mice. These findings suggest that Nur77’s effect on muscle growth is intrinsic to its expression in differentiating myofibers, and not dependent on its expression in myogenic stem cells. To determine the importance of *Nur77* expression in muscle accretion in mature mice, we generated an inducible-, muscle-specific, Nur77-deficient mouse model. We demonstrated that tamoxifen-induced deletion of *Nur77* in 3-month-old mice reduced myofiber size. This change was accompanied by increased activity of Smad2 and FoxO3, two negative regulators of muscle mass. The role of Nur77 in muscle growth was further elaborated in the cardiotoxin-induced muscle regeneration model. Compared to wildtype mice, regenerated myofibers were smaller in *Nur77*^*-/-*^ mice. However, when normalized to saline-injected muscle, the recovery of sarcoplasmic area was comparable between *Nur77*^*-/-*^ and wildtype mice. These findings suggest that Nur77 deficiency compromises myofiber growth, but not the regenerative capacity of myogenic progenitor cells. Collectively, the findings presented here demonstrate Nur77 as an important regulator of muscle growth both during prenatal and postnatal myogenesis.

## Introduction

Muscle wasting is a prevalent problem in disuse, diabetes, cancer cachexia, glucocorticoid excess, HIV, and aging. Skeletal muscle is the dominant site of insulin- and exercise-stimulated glucose disposal and a major target of insulin-sensitizing anti-diabetic medications. Reduced muscle mass impairs ambulatory function, stability, and systemic glucose metabolism. Optimizing muscle mass therefore has the potential to improve glycemic control, prevent disability, and improve quality of life. Uncovering regulatory pathways that control physiological muscle growth provides the basis for understanding and potentially reversing the pathological mechanisms of muscle wasting.

Skeletal muscle originates from mesodermal structures known as somites, which mature into dermomyotome and myotome that contain committed muscle stem cells. The differentiation of these myogenic progenitor cells into mature myotubes and myofibers is controlled by a cadre of transcription factors, including the paired-homeobox factors Pax3 and Pax7, and the myogenic regulatory factors (MRFs) Myf5, MyoD, myogenin, and Myf6 [1]. In mice, myotubes are detected as early as E12 [2]. These primary myotubes become the scaffold upon which perinatal myoblasts form secondary myotubes (by E16) [2,3]. The full complement of muscle fibers is achieved by birth or within one week after birth [4,5]. Postnatally, muscle growth occurs largely through muscle hypertrophy rather than hyperplasia [5]. Up until postnatal day 21 in mice, this process is mediated by robust satellite cell (muscle stem cell that resides beneath the basal lamina) proliferation and fusion with existing myofibers, thereby increasing the number of myonuclei per myofiber and the associated myofiber volume. Subsequent muscle hypertrophy in adulthood occurs with increases in myofiber volume without further accretion of myonuclei, effectively increasing the myonuclear domain [5,6].

Adult muscle mass is determined by the efficiency of developmental myogenesis as well as extrinsic influences including exercise, innervation, nutrient abundance, and multiple endocrine and paracrine growth factors. Many of these inputs converge on common intracellular pathways that control muscle mass. The most recognized pathway—the Akt/mTor/S6 signalling cascade, increases protein translation to promote muscle hypertrophy [7,8]. Just as importantly, calcium-dependent signaling regulates many aspects of muscle growth, including calcineurin-mediated cell fusion [9–11]. The effect of growth promoting pathways is counterbalanced by growth-limiting factors including TNFα, TNF-like weak inducer of apoptosis, myostatin, and glucocorticoid [12–16]. These signals stimulate the activities of transcription factors Smad2/3 and Forkhead box O transcription factors that result in proteolysis and muscle atrophy [17,18]. In addition, extensive crosstalk and feedback exists at different levels of these signaling cascades, the balance of which determines the net effect on muscle mass and myofiber size.

The Nr4a family of orphan nuclear receptors includes three highly conserved, homologous, and partially redundant members (Nr4a1, 2, and 3). As immediate-early genes, the expression of these receptors is upregulated acutely by a myriad of signals including cAMP, growth factors, mechanical stress, calcium, and cytokines [19]. Unlike canonical nuclear receptors, the putative ligand-binding domains of these receptors are blocked by bulky hydrophobic residues and cannot accommodate ligands [20,21]. Instead, the activities of these receptors are controlled by transcript abundance and post-translational modifications. In skeletal muscle, Nr4a1 –also known as Nur77 –is the most highly expressed Nr4a isoform and is preferentially expressed in fast-twitch muscle fibers [22]. We and others previously showed that *Nur77* expression is robustly induced by β-adrenergic stimulation and physical exercise [22–26]. *Nur77* expression directs the transcription of a battery of genes that control glucose uptake, glycolysis, and glycogenolysis [22]. Global deletion of *Nur77* predisposes mice to diet-induced obesity and insulin resistance [27]. On the other hand, constitutive overexpression of *Nur77* in skeletal muscle increases oxidative metabolism [28]. These results establish Nur77 as an important regulator of glucose metabolism in skeletal muscle.

In addition to its role in controlling glucose metabolism, we recently showed that adult mice with genetic deletion of *Nur77* in skeletal muscle exhibit reduced muscle mass and myofiber cross-sectional area (CSA) [29]. The reduction in muscle size was mediated by changes in the expression of *Igf1*, *myostatin*, *Trim63 (MuRF1)* and *Fbxo32 (MAFBx)* as well as signaling changes in the Akt/mTor/S6, Smad2/3, and FoxO pathways. Using primary myoblasts, we further showed that Nur77-directed changes in myotube caliber occur in both Igf1-dependent and independent pathways. What remained unknown, however, was whether the reduction in muscle mass reflects a defect in embryonic myogenesis or postnatal muscle growth. Using complementary genetic mouse models, we show here that *Nur77* expression in differentiating myofibers regulate muscle growth throughout development and in young adulthood in mice.

## Materials and methods

### Immunostaining of cryosections

Freshly isolated skeletal muscle was frozen in liquid nitrogen-chilled isopentane. 10μm sections were used for all immunostaining. Myofiber cross-sectional area and fiber composition were determined as previously described [29]. Primary antibodies were purchased from Developmental Studies Hybridoma Bank (type 1 fiber—A4.84, type 2a fiber—SC-71, type 2b fiber—BF-F3, and type 2d fiber– 6H1) and Sigma (laminin—L9393, fast myosin—MY32). A4.84 was also used for the detection of primary myofibers in E18.5 embryos. Secondary antibodies were purchased from Southern Biotechnology (anti-mouse IgM-FITC– 1021–02, anti-mouse IgG-555–1030–32) and ThermoFisher Scientific (anti-rabbit IgG-Alexa Fluor 350 –A11046). Staining of E18.5 immunosections were modified as follows. Slides were thawed on ice for 10 min and then fixed in 2% PFA at room temperature for 10 min. After washing in PBS, the sections were permeabilized with 0.1% Triton X-100 for 5 min, blocked in 15% horse serum in 0.1% Triton/PBS for 1 hour, and then incubated with primary antibodies in blocking buffer overnight at 4C. Slides were mounted with Prolong Gold Antifade Mountant (ThermoFisher Scientific), and visualized with the Leica DMI6000B inverted microscope and the Zeiss Axio Observer Inverted fluorescent microscopes and color CCD camera.

### Animal studies

The global and muscle-specific Nur77^-/-^ (mKO) mice have been described previously [22,29]. The inducible muscle-specific Nur77 knockout mice (subsequently referred to as imKO) were derived by introducing the HSA-MCM transgene into the floxed-Nur77 mouse. The HSA-MCM transgene consists of the human α-skeletal actin promoter driving the expression of the MerCreMer fusion gene, which includes a mutated estrogen receptor ligand-binding domain on each terminus of the Cre recombinase gene [30]. The HSA-MCM mice have been bred into the C57BL/6J background for 6 generations prior to mating with the floxed-Nur77 mouse [31]. Genotyping was performed by PCR amplification of a ~750bp product, using the following primers: Forward 5’ GGC ATG GTG GAG ATC TTT GA 3’, Reverse 5’ CGA CCG GCA AAC GGA CAG AAG 3’. The imKO mouse was injected at 3 months of age with tamoxifen 2mg/day by intraperitoneal route for 5 days, followed by a minimum of 10 days of washout period. Tamoxifen was prepared by mixing 150μl of 80mg/ml solution (Sigma T5648, dissolved in ethanol) with 850μl of sunflower seed oil. The suspension is vortexed briefly, then sonicated at 37C for 20 minutes. Tissue-specific Nur77 excision was confirmed by PCR amplification of isolated DNA, using primers F_1_ 5’ TGA CAC CCT CAC ACG GAC AA 3’, F_2_ 5’ ATG CCT CCC CTA CCA ATC TTC 3’, and R 5’ CCA GTA CAT AGA GGA TGC TTG TT 3’. Tamoxifen-induced Cre-mediated excision of the floxed-Nur77 cassette yields a 313bp F_1_-R amplicon, whereas the unexcised Nur77 allele produces a ~250bp F_2_-R amplicon. Mice were fed *ad libitum*, maintained on a 12-h light-dark cycle, and monitored at least once daily by staff from the Animal Care Facility at The Saban Research Institute. Mice were age and gender-matched for all experiments. Mice were euthanized with isoflurane prior to tissue isolation. This study was carried out in strict accordance with the recommendations in the Guide for the Care and Use of Laboratory Animals of the National Institutes of Health. The protocol was approved by the Children’s Hospital Los Angeles Institutional Animal Care and Use Committee. All efforts were made to minimize suffering.

### RNA and protein analyses

Total RNA preparation and quantitative real-time PCR were performed as described [22]. Expression was normalized to *36b4* expression. Primer sequences are as previously described [29]. Frozen muscle was homogenized in ice-cold Tris-Triton buffer (10mM Tris, pH 7.4, 100mM NaCl, 1mM EDTA, 1mM EGTA, 1% Triton X-100, 10% glycerol, 0.1% SDS, and 0.5% deoxycholate) with 1mM PMSF, 1X cOmplete protease inhibitor and PhosSTOP inhibitor from Roche) with a motorized Teflon pestle. Primary and secondary antibodies used were described previously [29]. Antibody binding was detected by ECL Plus (Pierce), using ImageQuant LAS4000 (GE) for image capture. Fiji was used for quantification of band intensity.

### Cardiotoxin injection

Mice were anesthetized with isoflurane. The hindlimbs were shaved and sterilized with alcohol wipe. 40μl of normal saline or 10μM cardiotoxin (Sigma C9759) was injected into the tibialis anterior (TA) muscle. 100μl of buprenorphine (0.025mg/ml) was injected subcutaneously for analgesia. All mice regained full-mobility without limping after recovery from anesthesia. Research staff monitored the mice daily for signs of distress, including ruffled fur, limping, and hunched posture. Mice exhibiting persistent distress (greater than 24 hours) were euthanized by CO_2_ asphyxiation. In all, cardiotoxin was injected into 92 mice, none required premature anesthesia, and 2 died prior to the end of the experiments. Mice were euthanized 3 to 15 days after cardiotoxin injection.

### Proliferation of myogenic progenitor cells

We injected bilateral TA muscles with cardiotoxin as described above. Muscle was harvested 3 days after cardiotoxin injection. BrdU 1mg was injected intraperitoneally 16h prior to muscle isolation. The methods for purification of myogenic progenitor cells were previously described and modified as below [32–34]. TA muscles were dissected into ice-cold HEPES-buffered 10% FBS/DMEM, minced, and gently agitated in sterile-filtered digestion buffer (0.2% collagenase B [Roche 11088823103], 0.16U/ml Dispase [Sigma D4818], 10mM HEPES in 10% FBS/DMEM) at 37C for 30 min. The muscle slurry was then triturated slowly through a 20-gauge needle 10 times and incubated at 37C for another 15 min. After another round of trituration, the muscle slurry was diluted in 10-volumes of 2% FBS/DMEM and passed through a 70μm mesh filter on ice. The suspension was spun down twice at 500 *g* and washed in 2% FBS/PBS. Cells were blocked in anti-mouse CD16/32 antibody (Biolegend 101320) for 15 min and incubated with the following antibodies for 15 min on ice in the dark: BV421 anti-rat CD31 (BD Biosciences 563356), PE anti-mouse Itga7 (Miltenyi 130-102-716), BV605 anti-rat CD45 (Biolegend 103139), BUV395 anti-rat Sca1 (BD Biosciences 563990), APC/Cy7 anti-rat CD11b (BD Biosciences 557654). A subset of cells was incubated with the corresponding isotype control antibodies: BV421 rat IgG2a (BD Biosciences 562602), PE mouse IgG1 (Miltenyi 130-098-845), BV605 rat IgG2b (Biolegend 400649), BUV395 rat IgG2a (BD Biosciences 563556), and APC/Cy7 rat IgG2b (Biolegend 400623). BrdU-negative control cells were isolated in parallel from wildtype and Nur77-null mice (n = 2 per genotype) that were not injected with BrdU. The cells were stained with anti-BrdU antibody following instructions from the BD Pharmingen^™^ APC BrdU Flow Kit. Cells were analyzed on the BD LSR II flow cytometer using FACSDiva software (version 6.1.2).

### Hematoxylin and eosin staining

10μm frozen sections were thawed and hydrated in water for 5 min, stained in Harris Hematoxylin for 5 min, rinsed in water, then differentiated in 0.5% HCl in 70% EtOH (10 dips). After rinsing in water, the sections were blued in 0.1% NaHCO_3_ for 30 sec, rinsed again, and incubated in 0.5% eosin in 0.5% acetic acid/70% EtOH for 2 min. After further rinsing, sections were dehydrated in ascending concentrations of EtOH (50%, 70%, 80%, 95%, and 100% EtOH), cleared in xylene, and then mounted with Permount mounting media. Slides were imaged on the Leica DMI6000B inverted microscope equipped with a color CCD camera.

### Statistical analysis

Student t-test was used to determine statistical significance. Error bars represent standard errors of the mean. Statistical significance between mean fiber-size was determined using unpaired t-test with Welch’s correction.

## Results

### Muscle Nur77 deletion reduces myofiber size by 3 weeks of age in mice

**Fig 1:**
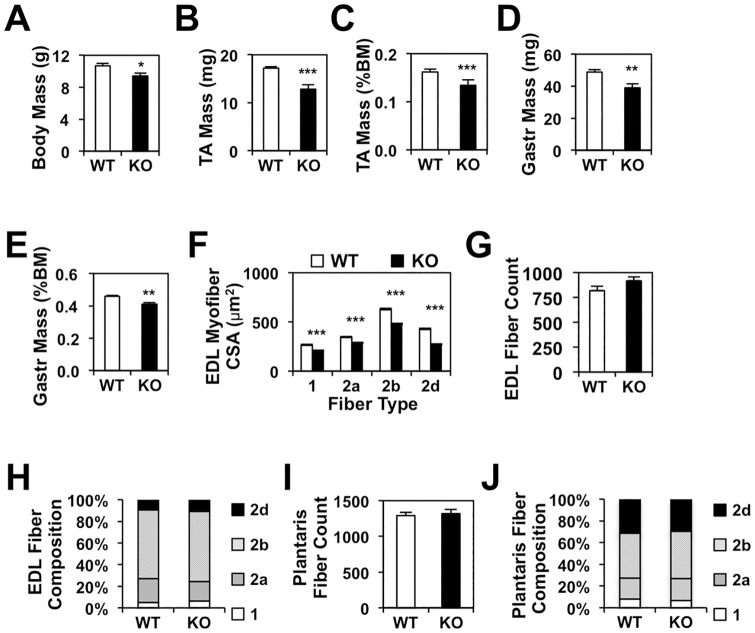
Nur77 deficiency reduces myofiber size by 3 weeks of age. (A-E) Body and muscle mass in 3-week-old global *Nur77*^*-/-*^ mice. N = 10–14. (F-H) Mean cross-sectional area (CSA), mean fiber count, and fiber composition of EDL muscle in wildtype and *Nur77*^*-/-*^ mice. N = 6. (I-J) Mean fiber count and fiber composition of plantaris muscle (N = 6–7). *P<0.05, **P<0.01, ***P<0.001. WT—wildtype, KO—knockout, TA—tibialis anterior, Gastr—gastrocnemius, BM—body mass, EDL—extensor digitorum longus.

We previously demonstrated that adult mice with *Nur77* deletion exhibited reduction in muscle mass and myofiber size [29]. It remained unclear, however, whether the muscle hypotrophy was attributable to defects in developmental myogenesis or muscle growth in adulthood. To investigate the role of Nur77 in muscle development, we first analyzed muscle fibers of 3-week-old global *Nur77*^*-/-*^ mice (postnatal day 22). In *Nur77*^-/-^ pups, we observed a 12% reduction in body mass, with concordant decreases in both absolute and normalized mass of the tibialis anterior (TA) and gastrocnemius muscles (Fig 1A–1E). Quantification of the cross-sectional area (CSA) of individual myofibers identified 17–36% reductions across all fiber types in the Nur77-deficient extensor digitorum longus (EDL) muscle (Fig 1F). The mean myofiber abundance and fiber composition were unchanged (Fig 1G–1J).

**Fig 2:**
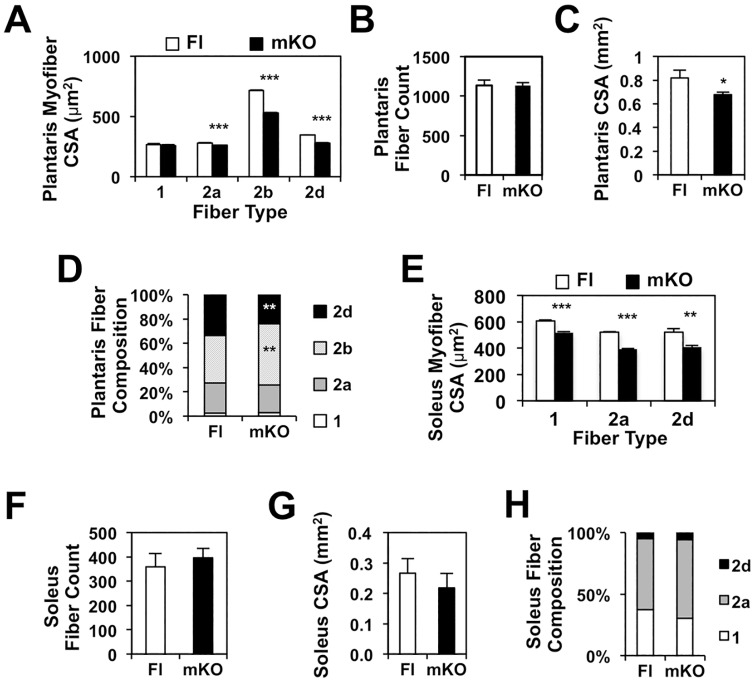
Muscle-specific Nur77 deletion is sufficient to reduce myofiber size in 3-week-old mice. Mean myofiber CSA, fiber count, mid-muscle CSA, and fiber composition of plantaris (A-D) and soleus muscles (E-H). N = 5–6 for plantaris, 4–6 for soleus. *P<0.05, **P<0.01, ***P<0.001. Fl—floxed-Nur77 control, mKO—muscle-specific Nur77 knockout.

The global Nur77^-/-^ mice lack Nur77 not only in differentiated myofibers but also in other cell types within the skeletal muscle that may contribute to myogenic differentiation, including the myogenic progenitor cells, mesoangioblasts, pericytes, and myoendothelial cells [35–37]. To determine if Nur77-mediated muscle growth is intrinsic to its expression in the myofibers or is dependent on the myogenic stem cell (MSC) compartment, we examined myofiber size in the myofiber-specific *Nur77*-deficient (mKO) mouse model. In the mKO mouse, Cre recombinase mediated excision of *Nur77* is under the control of the muscle creatine kinase (MCK) promoter, which was previously shown to be active only in differentiated multinucleated myotubes but not in myoblasts [29,38]. In the plantaris muscle of the 3-week-old mKO mice, mean myofiber CSA was significantly reduced in the fast-twitch fiber types, with the effect most notable for the glycolytic type 2b fibers (Fig 2A). The total number of fibers remained unchanged (Fig 2B), rendering an overall reduction in the mean plantaris muscle CSA (Fig 2C). Fiber composition analysis revealed a small increase in type 2b fibers at the expense of type 2d fibers (Fig 2D). That the mKO model recapitulated our finding in the global *Nur77*^*-/-*^ mice therefore supports our hypothesis that Nur77’s effect on muscle size is intrinsic to the differentiated myofibers and is independent of its expression in the MSC compartment.

We previously showed that the *Nur77* transcript is more abundant in fast-twitch muscles (e.g. EDL and quadriceps) than the slow-twitch muscle (e.g. soleus) [22]. To determine if deletion of *Nur77* also compromises the myofiber size of slow-twitch muscle, we analyzed the soleus muscle in the mKO mice. As shown in Fig 2E, the reduction of mean myofiber CSA of the soleus muscle was comparable to that observed in the fast-twitch plantaris muscle. The mean fiber abundance, soleus CSA, and fiber composition was not statistically different between control (floxed) and mKO mice (Fig 2E–2H). Type 2b fibers are not shown in Fig 2E and 2H because the slow-twitch soleus muscle lacks the fast-twitch glycolytic type 2b myofibers. These findings indicate that Nur77 is an important mediator of myofiber size in both fast- and slow-twitch muscles.

### *Nur77* deletion reduces size of secondary myofibers of E18.5 embryos

**Fig 3:**
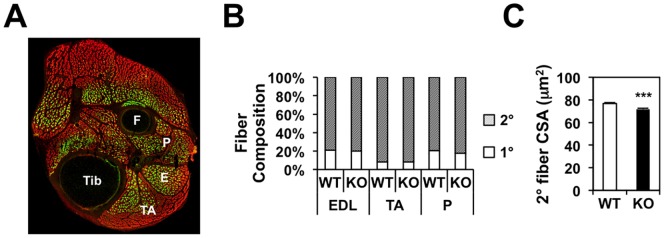
Nur77 deficiency reduces secondary myofiber size in E18.5 mouse hindlimb. (A) Representative immunostaining of E18.5 mouse hindlimb. Red—all myofibers (MY32-reactive), green—primary myofibers (A4.84-reactive). Secondary myofibers are identified as red fibers that were not green. F- fibula, Tib—tibialis, P—peroneus, E—EDL, TA—tibialis anterior. (B) Relative abundance of primary and secondary myofibers in E18.5 EDL, TA, and peroneus muscles. (C) CSA of secondary myofibers in the EDL. N = 7–8. ***P<0.001.

Having shown that *Nur77* deletion reduces myofiber size by postnatal day 22, we next examined if Nur77 deficiency compromises prenatal muscle development by E18.5. At this developmental stage, both primary and secondary myofibers are present, which subsequently give rise to adult slow- and fast-twitch myofibers, respectively. Using the hindlimbs of global *Nur77*^*-/-*^ embryos, we showed that the relative composition of primary and secondary myofibers is unchanged in the EDL, TA, and peroneus muscles of wildtype and global *Nur77*^-/-^ mice (Fig 3A and 3B). The total number of myofibers present in the EDL muscle was also unchanged (WT vs. KO: 787±37 vs. 733±53). We did observe a 7% decrease in the size of secondary myofibers, however (Fig 3C). These results indicate that Nur77’s regulation of muscle growth begins during developmental myogenesis and is detectable by E18.5 in mice.

### Acute *Nur77* deletion in adult mouse muscle reduces myofiber size

**Fig 4:**
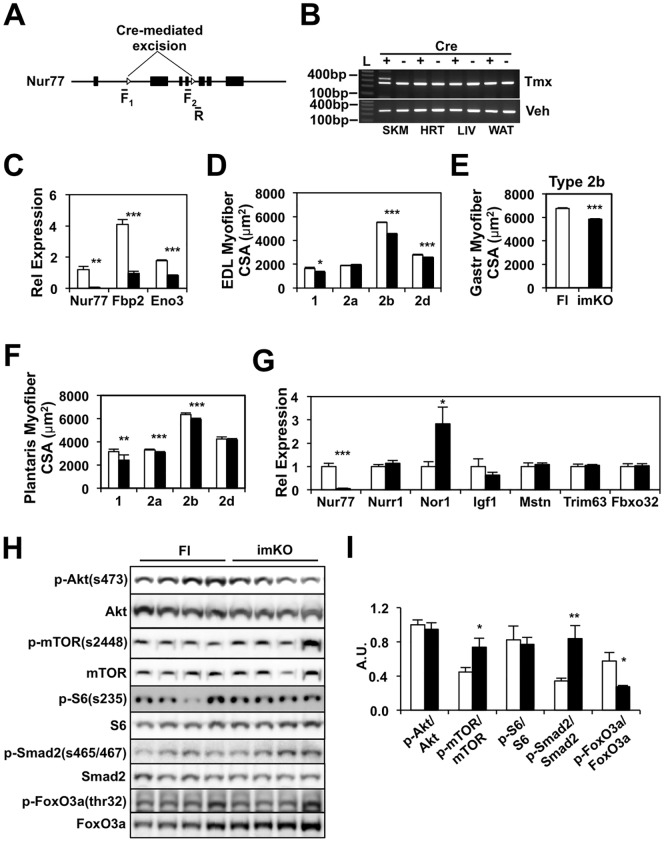
Characterization of the inducible, muscle-specific, Nur77-null (imKO) mouse. (A) Schematic of the floxed-Nur77 allele. Open triangle—LoxP site. F_1_, F_2_, and R denote forward and reverse genotyping primers. (B) Confirmation of tamoxifen- and muscle-specific deletion of Nur77 by PCR genotyping. Upper panel—mice injected with tamoxifen (Tmx); lower panel—mice injected with vehicle (Veh). L—ladder, SKM—skeletal muscle, HRT—heart, LIV—liver, WAT—white adipose tissue. (C) Expression of *Nur77* and its target genes in quadriceps muscle of control (Fl) and imKO mice one week after tamoxifen injection. N = 4. (D-F) Mean myofiber CSA of EDL, gastrocnemius, and plantaris muscles. N = 6–8. (G) Gene expression from white quadriceps of 6-month-old Fl and imKO mice. (H) Immunoblot analysis of EDL lysate. (I) Quantitation of immunoblot. N = 7. White bars denote floxed controls, black bars denote imKO mice. **P<0.01, **P<0.01, ***P<0.001.

Having demonstrated that *Nur77* expression is important in myofiber growth during developmental myogenesis and early postnatal life, we next proceeded to test the hypothesis that Nur77 also controls muscle accretion in the young adult mouse. To isolate the effect of Nur77 during adulthood and to avoid the confounding effect of developmental muscle hypotrophy in the constitutive Nur77 knockout models, we generated the tamoxifen-inducible, muscle-specific Nur77 knockout mouse (imKO). We introduced the inducible human skeletal actin (HSA-MCM) Cre driver allele into the floxed-Nur77 mouse (Fig 4A) [30,31]. The HSA-MCM Cre allele is expected to be active in myoblasts and differentiated skeletal muscle, but not in the heart or smooth muscles [30,39]. Tamoxifen, required for the activity of the MCM-Cre recombinase, was injected daily for five days into 3-month-old mice. Muscle-specific Cre-mediated excision of exons 2 through 4 of the *Nur77* gene was validated by PCR (Fig 4B). Tamoxifen-induced excision of the floxed-Nur77 exons was detectable in skeletal muscle (upper band in second lane of top panel of Fig 4B), but not in the heart, liver, or white adipose tissue. The presence of the lower band suggests incomplete excision of the floxed exons. We did not detect background recombination in muscle of vehicle-injected imKO mice (bottom panel of Fig 4B). However, to equalize the off-target effects of tamoxifen, subsequent studies were performed by administering tamoxifen to floxed-Nur77 mice with (imKO) or without (floxed-Nur77 only) the HSA-MCM-Cre transgene. The expression of *Nur77* and its target genes *Fbp2* and *Eno3* was reduced in skeletal muscle of the imKO mice, providing evidence for decreased Nur77 activity (Fig 4C and 4G). Having validated the tissue-specificity and loss of Nur77 function in the imKO mouse model, we next evaluated whether muscle mass or myofiber size was altered by 6 months of age (tamoxifen was injected at 3 months). As shown in Table 1, inducible *Nur77* deletion in adult mice did not alter body weight, length, fat mass, or muscle mass. However, we detected a significant reduction in mean myofiber CSA in the fast-twitch glycolytic type 2b myofibers (Fig 4D–4F: EDL 17%, gastrocnemius 13%, and plantaris 7%), indicating that in young adult mice, *Nur77* expression is an important contributor to muscle growth. The fiber composition and total fiber count of the EDL and plantaris muscles were unchanged (data not shown). We did not detect statistically significant differences in the cross-sectional area of these muscles (data not shown), however. We speculate that the latter result was due to subtle differences in the depths of the cryosections, which may be sufficient to obscure small reductions in the cross-sectional area of the muscles.

**Table 1:** Characteristics of imKO mice.

	Weight (g)	Length (cm)	WAT (mg)	WAT (%BW)	TA (mg)	TA (%BW)	Gastr (mg)	Gastr (%BW)
Fl	23.9±0.5	9.4±0.1	294±34	1.2±0.1	39±1	0.16±0.00	123±4	0.51±0.01
imKO	25.2±0.6	9.5±0.1	299±66	1.2±0.2	40±1	0.16±0.00	124±4	0.49±0.01

WAT—parametrial adipose tissue. Values represent mean ± standard error. N = 8 to 9.

To identify the mechanism behind the reduction in myofiber size in the imKO mice, we examined gene expression and signaling pathways that were altered in constitutive mutant Nur77 mice [29]. Unlike our findings in the *Nur77*^*-/-*^ mice, we did not detect significant differences in the expression of *Igf1* and growth-limiting genes such as *myostatin* (*Mstn*), *Trim63*, and *Fbxo32* in imKO muscle (Fig 4G). We did observe an upregulation of *Nor1*, but not *Nurr1* (two other members of the NR4A family) in response to *Nur77* deletion (Fig 4G), consistent with past reports of functional redundancy between Nur77 and Nor1 [22,40–42]. Immunoblot analysis did not reveal changes in Akt or S6 signaling, although mTor phosphorylation at Ser2448 was increased in the imKO mice (Fig 4H and 4I). The mTor complex coordinates a myriad of pathways in skeletal muscle, including lipid metabolism, mitochondrial metabolism, autophagy, and protein synthesis [43]. In the absence of changes in p-S6(ser235) (substrate of the mTor target S6K and a mediator of ribosomal biogenesis), the increase in p-mTor(ser2448) may represent an adaptive response to the reduction in myofiber size, and/or activation of other mTor-regulated pathways. Based on our previous work, we also examined protein activities downstream of myostatin and FoxO3 signaling. As shown in Fig 4H and 4I, ser465/467 phosphorylation of Smad2 was increased, whereas thr32 phosphorylation of FoxO3a was reduced in imKO muscle. Collectively, these changes are expected to limit muscle growth in the imKO mice.

### *Nur77* expression is not essential for muscle regeneration

**Fig 5:**
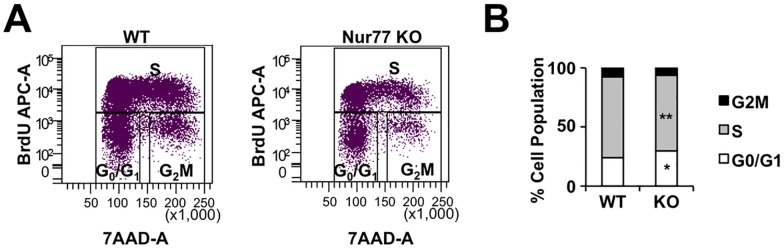
Cell cycle analysis of *Nur77*^*-/-*^ myogenic stem cells. (A) Measurement of wildtype and Nur77 knockout cells incorporating BrdU as a function of total DNA content. (B) Relative proportion of cells in different phases of cell cycle. N = 5. *P<0.05, **P<0.01.

We previously identified Nur77 as a transcriptional regulator of *Igf1* expression in skeletal muscle [29]. Based on Igf1’s established role in muscle regeneration, we next sought to determine the importance of *Nur77* expression in cardiotoxin-induced muscle regeneration. We first investigated whether *Nur77* expression is an important determinant in the proliferation of myogenic stem cells (MSCs) during muscle regeneration. For this study, we used global *Nur77*^*-/-*^ mice to eliminate *Nur77* expression in the MSCs. We injected cardiotoxin into the TA muscle and isolated MSCs by collagenous digestion and fluorescence-activated cell sorting three days later. MSCs were defined as cells with low CD11b, CD45, Sca1, and CD31 expression but high integrin alpha-7 (Itga7) expression [33,34]. Cell cycle analysis was performed to define the population of proliferating MSCs. In *Nur77*^*-/-*^ mice, we observed a small increase in the G_0_/G_1_ population (WT vs. KO: 23.9% vs. 29.3%) and a reduction in the percentage of MSCs in the S-phase (WT vs. KO: 68% vs. 63.4%) (Fig 5). This finding suggests that Nur77 deficiency impairs cell cycle progression and MSC proliferation, which may compromise muscle regeneration.

**Fig 6:**
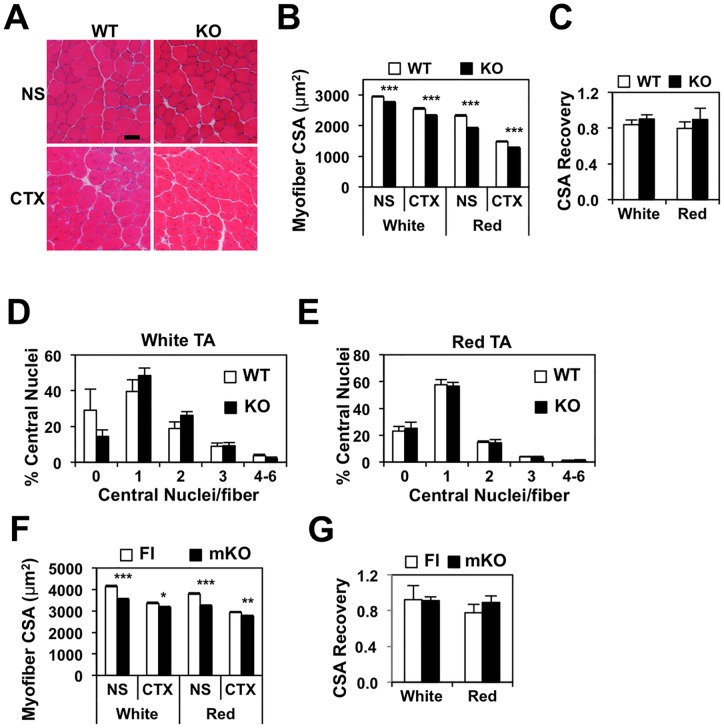
Cardiotoxin-induced muscle regeneration in Nur77-deficient mice. (A) H&E stains of cryosections from TA muscle isolated 15 days after normal saline (NS) or cardiotoxin (CTX) injection. Scale bar = 500 μm. Mean myofiber CSA, recovery of myofiber CSA, and percentage of myofibers with central nuclei from global Nur77 KO (B-E) and mKO (F-G) mice. N = 6–7 for B-E, 5–6 for F-G. *P<0.05, **P<0.01, ***P<0.001.

To determine if the small reduction in MSC proliferation was sufficient to impact muscle regeneration after cardiotoxin-injury, we analyzed the morphology of TA muscle 15 days after saline or cardiotoxin injection. We analyzed the myofiber size of the red (deep) and white (superficial) TA separately, due to intrinsic differences in fiber composition and size between these muscle compartments. Irrespective of genotype, the regenerated myofibers—marked by central nucleation—were expectedly smaller compared to control myofibers (Fig 6A and 6B). Myofiber size of the knockout muscle was smaller than its wildtype control in both saline- and cardiotoxin-injected TA. However, when the mean myofiber CSA of the cardiotoxin-injected TA was normalized to its saline-injected counterpart, we observed a comparable extent of myofiber recovery (Fig 6C). We also analyzed the frequency of central nucleation in the nascent myofibers, as a measure of myonuclear fusion efficacy. As shown in Fig 6D and 6E, Nur77 deficiency had no effect on the formation of multinucleated myofibers. These findings indicate that despite the early reduction in MSC proliferation, Nur77 deletion did not impair the regenerative capacity of injured muscle.

To confirm that *Nur77* expression in the MSC compartment is not necessary for regeneration, we replicated the cardiotoxin injection in myofiber-specific *Nur77*^*-/-*^ mice (mKO) and collected the TA muscle 15 days post injection. Similar to our findings in the global knockout mice, we found that in both saline and cardiotoxin injected TA, mKO myofiber CSA was smaller compared to the floxed-Nur77 control, although the difference was not as striking in the CTX-injected muscles (Fig 6F). Nevertheless, the extent of CSA recovery was similar between control and mKO muscle, providing further evidence supporting Nur77’s role in muscle growth but not regeneration (Fig 6G).

## Discussion

We previously showed that *Nur77* expression in skeletal muscle is a determinant of muscle mass in mice. The time course of this regulation remained unknown, however. Here we showed that the reduction in myofiber size was evident in Nur77-deficient mice size as early as E18.5, but became more pronounced by 3 weeks of age, highlighting the importance of *Nur77* expression in both fetal and postnatal muscle growth. We further demonstrated in a novel, inducible model of Nur77 deficiency, that loss of Nur77 during adulthood is sufficient to compromise myofiber size, supporting our hypothesis that Nur77 mediates myofiber growth during physiological muscle accretion in adulthood. Muscle mass in the imKO mice was unaffected, however, in contrast to the ~ 10% decrease in muscle mass we observed in the mKO and global knockout mice [29]. We speculate that this difference underscores the importance of muscle *Nur77* expression during muscle development, which is unperturbed in the imKO mouse. We also showed in the cardiotoxin-induced muscle regeneration model, that *Nur77* expression is not required for myogenic differentiation, but is important in the growth of the regenerated myofibers. Finally, we demonstrated here that the effect of Nur77 on muscle growth is intrinsic to its expression in differentiated muscles, and not from its action in the myogenic stem cell compartment.

In our analysis of myofiber composition in 3-week-old Nur77-deficient mice, we observed a shift from type 2d to type 2b fibers in the plantaris muscle of mKO, but not global knockout mice. The mechanism that belies this difference is unknown. However, we speculate that *Nur77* expression in the MSC compartment in the mKO mice may promote an adaptive response to increase the abundance of glycolytic fibers. Although this finding is unexpected given Nur77’s role in glycolytic metabolism, it is in-line with our result from the muscle-specific Nur77-overexpressing transgenic mice [28,29]. We previously demonstrated that constitutive overexpression of Nur77 in skeletal muscle enhanced oxidative metabolism and shifted the expression of troponin I and myosin heavy chain toward the slow-twitch isotypes. At the level of fiber composition, we observed a trend toward type 2d fibers at the expense of type 2b fibers in the Nur77-overexpressing EDL muscle, which is concordant with the result of our loss-of-function studies here. That the magnitude of the fiber composition change was rather modest suggests that Nur77 is not a major determinant of slow- vs. fast-twitch fiber identity. Rather, it likely reflects an adaptive response to Nur77-mediated regulations in mitochondrial dynamics and is distinct from Nur77’s transcriptional regulation of glycolytic genes that we previously described [22,28].

Activation of TGFβ-Smads 2/3 and FoxO signaling pathways and upregulation of E3 ubiquitin ligases *Fbxo32* and *Trim63* have been identified as important mediators of proteolysis in muscle atrophy [15]. In the imKO muscle, we observed increased Smad2 phosphorylation and decreased FoxO3a phosphorylation, which would be expected to reduce muscle mass. The expression of *Fbxo32* and *Trim32* was unchanged, however. This result is not unexpected, however, given previous reports of discrepancy between the abundance of active Smads 2/3 and FoxO transcription factors and *Fbxo32* and *Trim63* mRNA levels in both human and mouse studies. Sartori et. al. demonstrated that Smads 2/3 induced muscle atrophy in adult muscles independently of the expression of *Fbxo32* and *Trim63* [44]. In denervation-induced muscle atrophy, *Fbxo32* mRNA level rises acutely after denervation, but returns to basal levels by day 14 despite persistent increase in FoxO3 activity [45]. More recently, O’Neill et. al. showed that muscle atrophy in the MIGIRKO (muscle-specific insulin receptor/IGF-1 receptor knockout) mouse was accompanied by increased nuclear FoxO1 and FoxO3 abundance but unchanged *Trim63* and *Fbxo32* mRNA levels [46]. These findings suggest that atrogene-mediated proteolysis may be an early, self-limited event in muscle atrophy. Indeed, the expression of atrogenes was either unchanged or actually reduced in muscles from human subjects with long-standing muscle wasting [47,48]. As muscle wasting is not reversed after normalization of atrogene expression, additional catabolic pathways must be activated to prevent the recovery of muscle mass. In the MIGIRKO mouse, muscle atrophy was attributed to FoxO-mediated activation of autophagy rather than its proteasomal activity. We hypothesize that changes in autophagy or other stress-responsive transcriptional programs regulated by Foxo3a [18,49–51] may also contribute to the reduction in myofiber size in the imKO mouse model.

Muscle growth is intimately dependent on nutrient availability. In skeletal muscle, coordination of metabolism and muscle growth is mediated by several proteins in the Igf1-Akt-mTor pathway. In skeletal muscle, Igf1 stimulates fatty-acid uptake, lipid synthesis, and glucose metabolism—prerequisites to meet the demand of membrane expansion in the growing muscle [52,53]. The serine/threonine kinase Akt—a critical nexus for Igf1 and insulin signaling, exerts its control on muscle growth and metabolism through Akt1 and Akt2, respectively [54–57]. mTor is another such pleotropic regulator in the skeletal muscle. mTORC1 positively regulates muscle mass by increasing the translational efficiency in both Igf1-dependent and independent pathways [58–61]. Reducing mTOR activity in skeletal muscle has also been shown to stimulate oxidative metabolism in mice and enhance life span in *Drosophila* [62,63]. We now present findings, in the context of our prior studies, to bring Nur77 into this family of molecular regulators that coordinate glucose metabolism and muscle growth.

Our work here establishes the basis for future studies into the importance of Nur77 in metabolism and maintenance of muscle mass in muscle wasting. Patients with denervation atrophy have profoundly reduced glycolytic enzyme activities in their skeletal muscle [64]. As well, patients with spinal cord injury develop muscle atrophy and are at increased risk of developing insulin resistance and impaired glucose metabolism [65,66]. We previously demonstrated in rodents that denervation led to abrupt downregulation of *Nur77* and its glycolytic target genes, and that Nur77-deficient mice are predisposed to developing diet-induced obesity and insulin resistance [22,27]. Here we present evidence that Nur77 is a mediator of myofiber growth during development and adulthood. These findings raise the possibility that overexpressing *Nur77* may mitigate muscle wasting and improve glucose metabolism. Studies in human subjects have also shown that Nur77 is one of the most robustly induced genes by intense cycling [23,25]. *Nur77* expression in this scenario is expected to stimulate glycolytic metabolism and increase ATP production. It is also plausible, however, Nur77 is upregulated to facilitate loading-induced muscle hypertrophy. Experimental models of overload may be used in the inducible model of Nur77-deletion (imKO mice) to elucidate the role of Nur77 in this process. Finally, preferential atrophy of type 2 (fast-twitch) glycolytic myofibers is observed in aging and Duchenne muscular dystrophy (DMD) [67,68]. As *Nur77* is selectively expressed in type 2 glycolytic myofibers, analysis of muscle *Nur77* expression in mouse models of DMD and sarcopenia will help determine whether Nur77 is a viable target in the management of muscle wasting in these conditions.
